# The Complete Mitochondrial Genome of *Aspidophorodon* (*Eoessigia*) *indicum* (Hemiptera: Aphididae: Aphidinae) and Insights into Its Phylogenetic Position

**DOI:** 10.3390/genes16080979

**Published:** 2025-08-20

**Authors:** Jiayu Ding, Xiaolu Zhang, Liyun Jiang, Gexia Qiao, Jing Chen

**Affiliations:** 1 State Key Laboratory of Animal Biodiversity Conservation and Integrated Pest Management, Institute of Zoology, Chinese Academy of Sciences, Beijing 100101, China; dingjiayu2023@ioz.ac.cn (J.D.); zhangxiaolu@ioz.ac.cn (X.Z.); jiangliyun@ioz.ac.cn (L.J.); 2 College of Life Sciences, University of Chinese Academy of Sciences, Beijing 100049, China

**Keywords:** Macrosiphini, repeat region, *Pterocomma* group

## Abstract

Background: *Aspidophorodon* Verma, 1967 (Macrosiphini: Aphidinae), is a genus within Aphididae (aphids) with ecological importance and a unique distribution, but there is a lack of mitogenomic data on the evolutionary relationships within this genus, hindering a comprehensive understanding of its evolutionary history. Methods: In this study, we present the complete mitochondrial genome sequence and features of *Aspidophorodon indicum* (David, Rajasingh & Narayanan, 1972) (Hemiptera: Aphididae) and further infer its phylogenetic position based on the complete mitochondrial genome sequence. Results: The complete mitochondrial genome of *A. indicum* is 17,161 bp in length, including 13 protein-coding genes, 22 transfer RNA genes, 2 ribosomal RNA genes, a control region, and a repeat region between *trnE* and *trnF*. Phylogenetic analyses based on complete mitochondrial genomes of Aphidinae indicated that the two constituent tribes, Macrosiphini and Aphidini, are monophyletic. *Aspidophorodon* was robustly clustered with the members of *Pterocomma* and *Cavariella*. Together, these three genera form the most basal clade within Macrosiphini. Conclusions: The complete mitogenome of *A. indicum* contains multiple conserved features relative to other aphids, including gene order, nucleotide composition, codon bias, and repeat region. The phylogenetic relationships within Macrosiphini reported here are consistent with previous studies. Our results provide new insights into the phylogenetic position of the genus *Aspidophorodon*.

## 1. Introduction

*Aspidophorodon* Verma, 1967 (Aphidinae: Macrosiphini), is an aphid genus characterized by individuals with a head containing three frontal processes; a wrinkled, reticulated, or sculptured dorsum that may feature papillate tubercles; and siphunculi that are long, spoon-shaped, broad at the base, and slightly swollen at the distal end, and that lack a flange [1]. Some species in this genus also have rows of long processes on thoracic notum and abdominal tergites. *Aspidophorodon* is distributed in Asia and North America and comprises two subgenera: the nominal subgenus and subgenus *Eoessigia* David, Rajasingh & Narayanan, 1972, which mainly feed on *Salix* Linnaeus, 1753 (Salicaceae) and plants within Rosaceae (*Cotoneaster* Medikus, 1789, *Potentilla* Linnaeus, 1753, etc.), respectively [2,3]. Ten of the fifteen known species in this genus are distributed exclusively in the Qinghai–Tibetan Plateau (QTP) and the Himalayas [1,2,3]. As a representative insect taxon in this region, *Aspidophorodon* holds significant value for the study of invertebrate diversification in the QTP–Himalayas region. Research on this genus has primarily focused on taxonomy. No studies have explored its phylogenetic relationships or evolutionary history.

*Aspidophorodon* (*Eoessigia*) *indicum* (David, Rajasingh & Narayanan, 1972) [4] is the type species of the subgenus *Eoessigia*. It is heteroecious and holocyclic, colonizing along the main veins of the upsides of *Cotoneaster* leaves and migrating to the undersides of the leaves of *Potentilla* from April to May [5,6]. *A. indicum* was once reported to be present only in India [7]. Recently, Xu et al. (2022) [1] recorded it in Xizang, China, for the first time.

To date, molecular data resources for the genus *Aspidophorodon* remain very scarce, with no mitochondrial genomic data available. Mitochondrial genomes have been increasingly used in aphid phylogenetic and population genetic studies [7,8,9]. The tribe Macrosiphini, to which *Aspidophorodon* belongs, is quite a large and heterogeneous group, comprising more than 30% of known aphid species [10]. So far, complete mitochondrial genomes have been reported for approximately 40 species of Macrosiphini. These mitogenome sequences range in size from 15 to18 kb. Notably, the species-specific repeat region located between *trnE* and *trnF* has been found in the mitogenomes of many macrosiphine aphids [11,12,13]. Using limited nuclear and mitochondrial genes, von Dohlen et al. (2006) [14] and Choi et al. (2018) [11] investigated the phylogenetic relationships of Aphidinae and Macrosiphini, respectively. However, neither study included samples from the genus *Aspidophorodon*, leaving its phylogenetic position unclear. Only in the phylogeny of Aphididae based on genes of *Buchnera* Munson, Baumann & Kinsey, 1991 [15], has *Aspidophorodon* been sampled. *Aspidophorodon* and *Capitophorus* van der Goot, 1913, were found to form a sister group, and together with *Pterocomma* Buckton, 1879, the three genera constituted the most basal lineage within Macrosiphini.

In this study, we obtained the first complete mitogenome sequence of the genus *Aspidophorodon*, *A. indicum*, along with the details of its mitogenomic structure, providing a valuable data resource with which to address the lack of mitogenomes in this genus. Based on mitogenome data, we reconstructed the phylogenetic relationships of Aphidinae and discussed the phylogenetic position of *Aspidophorodon*, contributing novel molecular evidence for understanding the evolution of this group.

## 2. Materials and Methods

### 2.1. Sample Collection and DNA Extraction

The aphid samples were collected from the upsides of leaves of *Cotoneaster glaucophyllus* Franchet, 1890, in Cuona, Xizang, China (27°50′24″ N, 91°46′12″ E), by Fangfang Niu on 3 June 2016. Approximately 30 aphids were collected and rapidly stored in 95% ethanol with a brush in the field. All samples were then preserved at −30 °C in the National Animal Collection Resource Center, Institute of Zoology, Chinese Academy of Sciences, Beijing, China (voucher no. 37232). The slide-mounted voucher specimens of apterous viviparous females were identified based on morphological characteristics (e.g., 6-segmented antennae; a median frontal tubercle that is slightly depressed in the middle; spinal processes that are only present on abdominal tergite VIII and conical in shape; and a dorsum of the head, thoracic nota, and abdominal tergites I–VII exhibiting semicircular and wavy structures) according to a taxonomic key [1]. Genomic DNA was extracted using the cetyltrimethylammonium bromide (CTAB) method.

### 2.2. Sequencing, Assembly, and Annotation

The mitogenome of *A. indicum* was sequenced using an Illumina NovaSeq 6000 platform (Biomarker Technologies Co., Ltd., Beijing, China). Approximately 20 Gb of raw data was generated. Quality control was conducted using Trimmomatic-0.39 [16], and 17 Gb of clean data was obtained. Mitogenome assembly was performed using NOVOPlasty v4.3.5 [17]. Preliminary annotation results were obtained using MITOS2 (2.1.9+galaxy0) [18] with the invertebrate mitochondrial genetic code. The positions of all genes were then manually corrected by aligning them with closely related aphid mitogenomes in MEGA-X v10.2.6 [19]. Protein-coding genes (PCGs) were further validated using ORF Finder (https://www.ncbi.nlm.nih.gov/orffinder/, accessed on 6 June 2025). The secondary structures of tRNAs were predicted and visualized using MITOS2 and VARNA v3-93 [20], respectively. The repeat units were identified through the Tandem Repeats Finder web server (https://tandem.bu.edu/trf/home, accessed on 6 June 2025) [21]. The annotated mitogenome sequence has been submitted to NCBI GenBank under accession number PQ468004. The mitogenomic structure was visualized through Proksee (https://proksee.ca/, accessed on 6 June 2025) [22].

### 2.3. Sequence Analyses

The nucleotide composition of the *A. indicum* mitogenome was obtained using PhyloSuite v1.2.3pre3 [23,24]. The composition skew of the mitogenome was calculated using the following formulae: AT skew = (A − T)/(A + T) and GC skew = (G − C)/(G + C) [25]. The relative synonymous codon usage (RSCU) of the PCGs was computed and depicted using PhyloSuite v1.2.3pre3. Signals of selective pressure were measured using the ratio of non-synonymous substitutions (Ka) to synonymous substitutions (Ks) for each PCG using DnaSP v6.12.03 [26], with *Adelges tsugae* (GenBank accession no. MT263947) taken as a reference. The sequence similarity of repeat units located in the control region and the repeat region was calculated using the Sequence Manipulation Suite (Version 2) (https://www.detaibio.com/sms2/, accessed on 9 June 2025) [27].

### 2.4. Phylogenetic Analysis

Twenty-seven species of Aphidinae, including *A. indicum*, which was sequenced and annotated in this study, were used to construct the phylogenetic trees. Four species from the subfamilies Calaphidinae and Chaitophorinae were chosen to be the outgroups based on previous phylogenetic studies on aphids [28,29,30]. All species used in the phylogenetic analyses, along with the accession numbers of their mitogenomes, are included in Table S1. The mitogenome sequences of all species are complete except for *Pterocomma pilosum* Buckton, 1879. Each gene was aligned using MAFFT v7.505 [31]. The aligned sequences of PCGs and RNAs were trimmed using trimAl v1.2 [32] and Gblocks 0.91b [33], respectively. Subsequently, we concatenated all 37 genes with a total of 14,341 bp using PhyloSuite v1.2.3pre3. The maximum likelihood (ML) tree was inferred using IQ-TREE v2.2.2.7 [34] with 1000 bootstrap replications, and the best partition and substitution models were selected with -m MFP+MERGE. For Bayesian inference (BI) analysis, PartitionFinder2 v2.1.1 [35] was used to evaluate the optimal partitioning scheme and the optimal model for each partition. The BI tree was constructed in MrBayes v3.2.7 [36]. Chains were run for 3,000,000 generations, with a sampling frequency of 100 and a relative burn-in of 25%. The phylogenetic tree was edited in Figtree v1.4.4 [37] and further embellished in iTOL (https://itol.embl.de/, accessed on 18 June 2025) [38].

## 3. Results and Discussion

### 3.1. Mitogenome Organization and Nucleotide Composition

**Figure 1:**
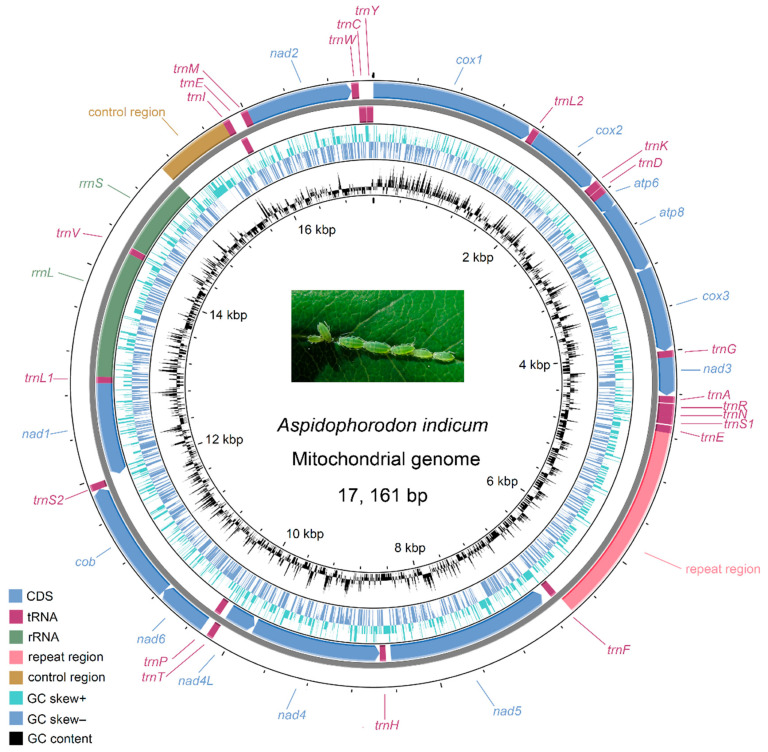
Circular map of the *Aspidophorodon indicum* mitogenome, showing the arrangement of 37 genes and two non-coding regions as well as variations in GC content and GC skew.

The complete mitogenome of *A. indicum* is a circular molecule of 17,161 bp, encoding 13 PCGs, 22 transfer RNA genes (tRNAs), and 2 ribosomal RNA genes (rRNAs). The gene order is conserved following the putative ancestral insect [39]. The organization of 37 genes and two non-coding regions, together with the GC content and GC skew along the mitogenome, is presented in Figure 1. Twenty-three genes are located on the majority strand (J-strand), while four PCGs (*nad5*, *nad4*, *nad4L*, and *nad1*), eight tRNAs (*trnF*, *trnH*, *trnP*, *trnL1*, *trnV*, *trnQ*, *trnC*, and *trnY*), and two rRNAs (*rrnL* and *rrnS*) are encoded on the minority strand (N-strand). There are two long non-coding regions in the *A. indicum* mitogenome. One is the control region located between *rrnS* and *trnI*, and the other is the repeat region located between *trnE* and *trnF*. Basic information on all the genes and non-coding regions is listed in Table 1, including strand orientation, positions, lengths, tRNA anticodons, the start and stop codons of the PCGs, and intergenic spacer lengths. The whole mitogenome consists of 45.9% A, 38.2% T, 10.5% C, and 5.4% G. It shows an obvious bias towards A + T (84.1%), similar to what is observed in other aphid species [9,13]. The *A. indicum* mitogenome is slightly A-skewed and moderately C-skewed, with a positive AT skew of 0.092 and a negative GC skew of −0.319 (Table 2).

### 3.2. Protein-Coding Genes

**Figure 2:**
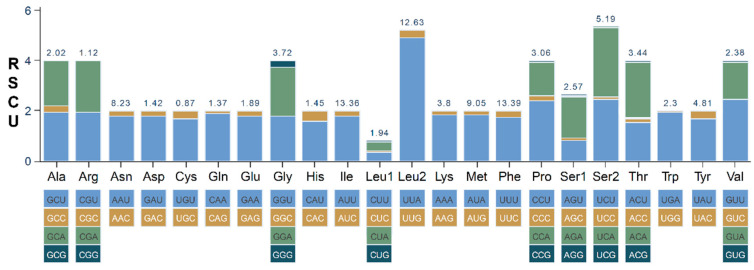
Relative synonymous codon usage (RSCU) of the *A. indicum* mitogenome, with amino acid frequencies indicated above.

The mitogenome of *A. indicum* includes 13 PCGs, ranging from 159 to 1731 bp in length. The A + T content of its PCGs is 82.3%. All the PCGs start with ATN and end with TAA, except for *cox1* and *nad4*, which end with a single T. RSCU values were calculated to measure the codon usage bias (Figure 2). The codons with an RSCU value > 1.0 were defined as abundant codons. The three most abundant codon families in the *A. indicum* mitogenome are Phe, Ile, and Leu (UUR), as observed in many aphids [9,40,41]. The Ka/Ks values of the PCGs are shown in Table 3. All the PCGs except *atp8* have Ka/Ks values below one, indicating purifying selection. The *atp8* gene is under positive selection, with the highest evolutionary rate: 1.2785.

### 3.3. Transfer RNAs and Ribosomal RNAs

**Figure 3:**
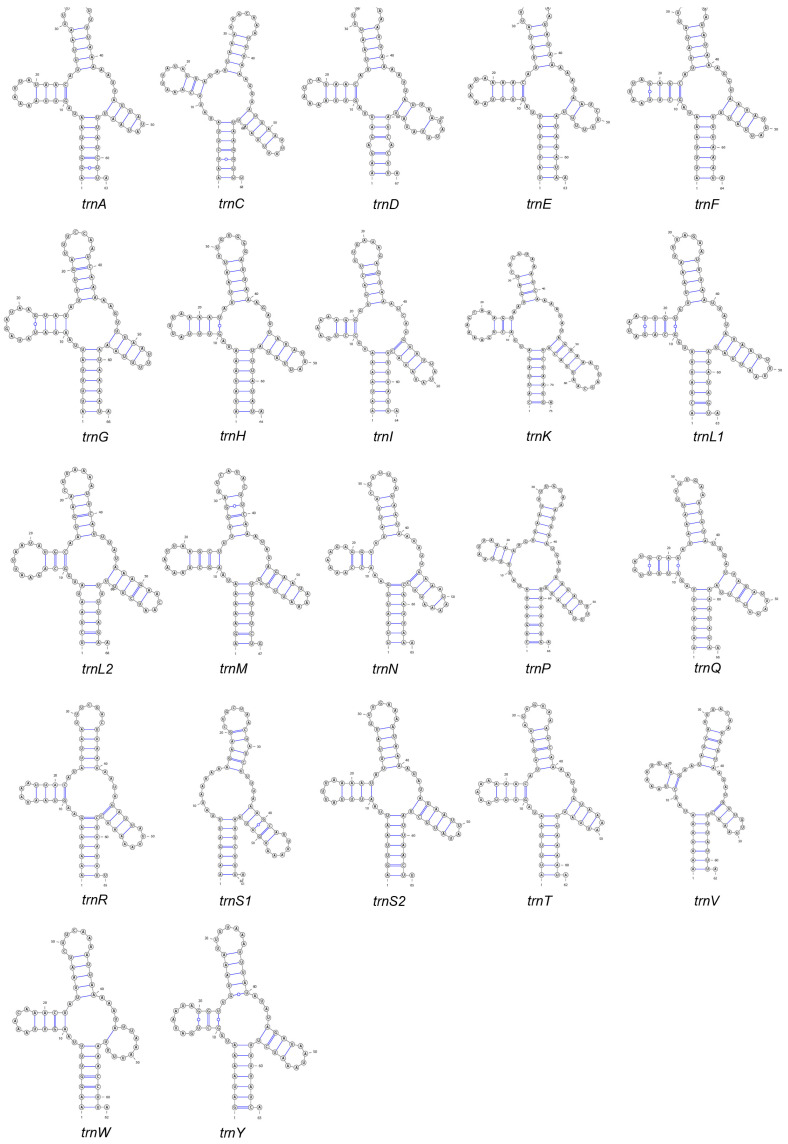
Secondary structures of tRNAs in the *A. indicum* mitogenome.

*A. indicum* has 22 tRNAs, ranging from 61 to 73 bp in length. These tRNAs exhibit an AT bias (85.2%), similar to the overall mitogenomic composition. The AT and GC skew values are 0.028 and 0.175, respectively (Table 1). Except *trnS1*, all the tRNAs are folded into typical clover-leaf secondary structures (Figure 3), consisting of four domains and a variable loop. *trnS1* lacks the dihydrouridine (DHU) arm, which is almost ubiquitous in insect mitochondrial genomes [42,43]. The mitogenome of *A. indicum* also contains two rRNAs. *rrnL* is 1263 bp long and positioned between *trnL1* and *trnV*, while *rrnS* is 770 bp long and located between *trnV* and the control region.

### 3.4. Control Region and Repeat Region

The control region is a long non-coding region believed to be involved in the initiation of DNA replication [44,45,46]. The control region in *A. indicum* is 1135 bp long, with an A + T content of 89.4%, and it is situated between *rrnS* and *trnI*. We found a tandem repeat sequence within the control region, consisting of a 107 bp repeat unit repeated 4.3 times.

In the mitogenome of *A. indicum*, the repeat region, which is specific to aphids, is located between *trnE* and *trnF*. The repeat region is 1158 bp long, with an A + T content of 90.0%. It contains a repeat unit that is 204 bp long and repeats 5.5 times. This repeat region may represent an additional origin of replication [46,47]. It has been found in different aphid subfamilies [13] and may have originated in the common ancestor of Aphididae [13,47,48]. The repeat sequences within the control region and the repeat region differ in terms of structural organization. Sequence comparison showed that their repeat units share only 35.29% nucleotide similarity, indicating distinct evolutionary origins [13,49].

### 3.5. Phylogenetic Relationships

**Figure 4:**
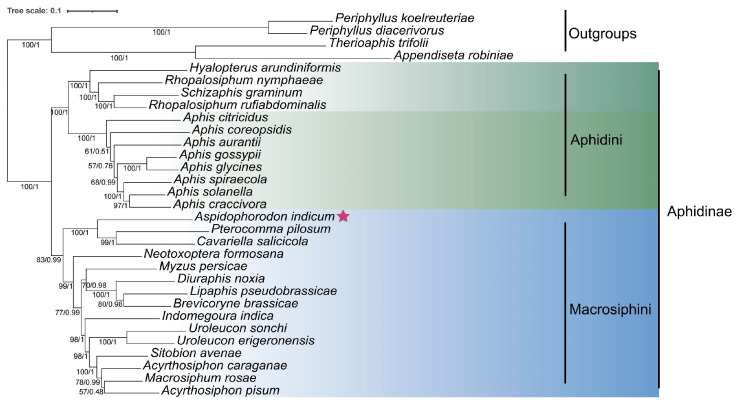
The phylogenetic tree of Aphidinae inferred from complete mitochondrial genomes. Values under branches indicate ML bootstrap values on the left and BI posterior probabilities on the right. The star in the figure indicates the position of *A. indicum* in the phylogenetic tree.

The phylogenetic relationships of Aphidinae were determined via the ML and BI methods based on the complete mitogenomes of *A. indicum* and 30 other species. The two trees exhibited nearly congruent topologies. Therefore, we present the tree, along with both bootstrap values and posterior Bayesian probabilities, in Figure 4. There was strong support for the subfamily Aphidinae being monophyletic. Within the Aphidinae, two tribes, Aphidini and Macrosiphini, were also monophyletic. The relationships within Macrosiphini observed in this study are generally consistent with the results reported by Choi et al. (2018) [11]. *A. indicum* was clustered with *Pterocomma pilosum* and *Cavariella salicicola* (Matsumura, 1917), and these three species formed the most basal clade of Macrosiphini. *Neotoxoptera* Theobald, 1915 was the second earliest-diverging lineage in Macrosiphini. The remaining species were split into two clades, one consisting of *Myzus* Passerini, 1860, *Diuraphis* Aizenberg, 1935, *Lipaphis* Börner, 1939, and *Brevicoryne* Das, 1915 and the other comprising *Indomegoura* Hille Ris Lambers, 1958, *Uroleucon* Mordvilko, 1914, *Sitobion* Mordvilko, 1914, *Acyrthosiphon* Mordvilko, 1914, and *Macrosiphum* Passerini, 1860.

Using multiple *Buchnera*-derived genes, Nováková et al. (2013) derived the phylogeny of Aphididae, in which the genus *Aspidophorodon* was placed as a sister to *Capitophorus*, and these two genera were firmly clustered with *Pterocomma* [15]. In the study by Choi et al. (2018) [11], *Capitophorus*, *Pterocomma*, and *Cavariella* were positioned in the “*Pterocomma* group”, which was placed as a sister to all the remaining species of Macrosiphini. In our study, based on complete mitochondrial genomes, *Aspidophorodon*, *Pterocomma*, and *Cavariella* were found to form a well-supported, basal monophyletic clade within Macrosiphini. It is worth noting that these three genera all feed on plants in the Salicaceae family [50]. Therefore, our mitogenomic phylogeny suggests that *Aspidophorodon* should also be a member of the “*Pterocomma* group”.

## 4. Conclusions

The complete mitogenome of *A. indicum*, representing the first mitogenome of *Aspidophorodon*, was generated through next-generation sequencing. We characterized the mitogenome architecture of *A. indicum* and found that it shares many significant features with other aphids, including in terms of gene order, nucleotide composition, codon usage bias, and the presence of a repeat region situated between *trnE* and *trnF*. Phylogenetic trees were constructed using all 37 mitochondrial genes, which revealed the phylogenetic relationships of Macrosiphini, aligning with previous studies in this respect. A close relationship was observed between *Aspidophorodon*, *Pterocomma*, and *Cavariella*, which together formed the basal lineage within Macrosiphini. The newly produced mitogenome sequence of *A. indicum* allows us to view the genus *Aspidophorodon* from a perspective differing from that of morphology-based taxonomy. There is a pressing need to acquire more aphid mitogenomes to enhance our understanding of the phylogeny and evolution of macrosiphine aphids.

## Figures and Tables

**Table 1:** Positions and basic features of genes and non-coding regions of the *Aspidophorodon indicum* mitogenome.

Gene	Strand	Position	Length (bp)	Anticodon	Start Codon	Stop Codon	Intergenic Nucleotides (bp)
*cox1*	J	1–1531	1531		ATA	T	0
*trnL2*	J	1532–1599	68	TAA			3
*cox2*	J	1603–2274	672		ATA	TAA	2
*trnK*	J	2277–2349	73	CTT			0
*trnD*	J	2350–2416	67	GTC			0
*atp8*	J	2417–2575	159		ATC	TAA	–20
*atp6*	J	2556–3209	654		ATT	TAA	–1
*cox3*	J	3209–3994	786		ATG	TAA	4
*trnG*	J	3999–4064	66	TCC			0
*nad3*	J	4065–4418	354		ATA	TAA	–1
*trnA*	J	4418–4480	63	TGC			–1
*trnR*	J	4480–4544	65	TCG			–1
*trnN*	J	4544–4608	65	GTT			–1
*trnS1*	J	4608–4668	61	GCT			7
*trnE*	J	4676–4738	63	TTC			0
Repeat region		4739–5896	1158				0
*trnF*	N	5897–5960	64	GAA			0
*nad5*	N	5961–7691	1731		ATT	TAA	362
*trnH*	N	8054–8117	64	GTG			0
*nad4*	N	8118–9426	1309		ATA	T	8
*nad4L*	N	9435–9725	291		ATG	TAA	1
*trnT*	J	9727–9788	62	TGT			2
*trnP*	N	9791–9856	66	TGG			1
*nad6*	J	9858–10,352	495		ATT	TAA	–1
*cob*	J	10,352–11,467	1116		ATG	TAA	11
*trnS2*	J	11,479–11,543	65	TGA			10
*nad1*	N	11,554–12,489	936		ATT	TAA	0
*trnL1*	N	12,490–12,554	65	TAG			0
*rrnL*	N	12,555–13,817	1263				0
*trnV*	N	13,818–13,879	62	TAC			10
*rrnS*	N	13,890–14,659	770				0
Control region		14,660–15,794	1135				0
*trnI*	J	15,795–15,858	64	GAT			–3
*trnQ*	N	15,856–15,921	66	TTG			5
*trnM*	J	15,927–15,993	67	CAT			0
*nad2*	J	15,994–16,971	978		ATA	TAA	–2
*trnW*	J	16,970–17,031	62	TCA			–8
*trnC*	N	17,024–17,091	68	GCA			4
*trnY*	N	17,096–17,160	65	GTA			1

**Table 2:** Nucleotide composition and skewness of the *A. indicum* mitogenome.

	T%	C%	A%	G%	A + T%	AT Skew	GC Skew
Whole mitogenome	38.2	10.5	45.9	5.4	84.1	0.092	–0.319
Protein-coding genes	47.3	9.5	35.0	8.1	82.3	–0.149	–0.080
1st codon positions	39.4	8.9	40.3	11.4	79.7	0.012	0.122
2nd codon positions	52.6	13.7	23.2	10.5	75.8	–0.388	–0.129
3rd codon positions	50.1	5.9	41.6	2.4	91.7	–0.092	–0.430
Protein-coding genes-J	42.1	12.4	38.6	6.9	80.7	–0.044	–0.286
Protein-coding genes-N	55.6	5.0	29.4	10.1	85.0	–0.308	0.336
tRNA genes	41.4	6.1	43.8	8.7	85.2	0.028	0.175
rRNA genes	45.8	5.0	38.8	10.5	84.6	–0.083	0.357
Control region	44.8	7.2	44.6	3.4	89.4	–0.002	–0.358
Repeat region	38.0	8.2	52.0	1.8	90.0	0.156	–0.640

**Table 3:** Ka, Ks, and Ka/Ks values of 13 protein-coding genes within the *A. indicum* mitogenome.

Gene	Ka	Ks	Ka/Ks
*apt6*	0.2062	0.6915	0.2982
*atp8*	0.4471	0.3497	1.2785
*cox1*	0.0528	0.7935	0.0665
*cox2*	0.0934	0.8912	0.1048
*cox3*	0.1259	0.6156	0.2045
*cob*	0.1114	0.7901	0.1410
*nad1*	0.1256	0.4042	0.3107
*nad2*	0.2001	0.611	0.3275
*nad3*	0.1768	0.5424	0.3260
*nad4*	0.1524	0.3251	0.4688
*nad4L*	0.1253	0.3344	0.3747
*nad5*	0.1459	0.3484	0.4188
*nad6*	0.2589	0.6453	0.4012

## Data Availability

The data that support the findings of this study are openly available in GenBank with accession number PQ468004.

## Supplementary Materials

The following supporting information can be downloaded at https://www.mdpi.com/article/10.3390/genes16080979/s1. Table S1: All aphid species used in the phylogenetic analyses.
