# Supplementary material for: The Complete Mitochondrial Genome of Aspidophorodon (Eoessigia) indicum (Hemiptera: Aphididae: Aphidinae) and Insights into Its Phylogenetic Position
Source: Genes (Basel). 2025 Aug 20;16(8):979. doi: 10.3390/genes16080979 (PMC12385917; doi:10.3390/genes16080979)
Supplement: Supplementary file 1 [file genes-16-00979-s001.zip › genes-3782478-supplementary.pdf]

**Table S1** All aphid species used in the phylogenetic analyses.

| Family | Subfamily | Tribe | Subtribe | Species | Length (bp) | Accession number |
|---|---|---|---|---|---|---|
| Aphididae | Aphidinae | Aphidini | Aphidina | *Aphis aurantii* Boyer de Fonscolombe, 1841 | 15296 | NC_052865 |
| | | | | *Aphis craccivora* Koch, 1854 | 15308 | NC_031387 |
| | | | | *Aphis citricidus* (Kirkaldy, 1907) | 16763 | NC_043903 |
| | | | | *Aphis coreopsidis* (Thomas, 1878) | 15623 | NC_068763 |
| | | | | *Aphis glycines* Matsumura, 1917 | 17954 | NC_045236 |
| | | | | *Aphis gossypii* Glover, 1877 | 15869 | NC_024581 |
| | | | | *Aphis solanella* Theobald, 1914 | 15331 | NC_068764 |
| | | | | *Aphis spiraecola* Patch, 1914 | 15465 | NC_053819 |
| | | | Rhopalosiphina | *Hyalopterus arundiniformis* Ghulamullah, 1942 | 15408 | OK274075 |
| | | | | *Rhopalosiphum nymphaeae* (Linnaeus, 1761) | 15594 | MN943499 |
| | | | | *Rhopalosiphum rufiabdominale* (Sasaki, 1899) | 15289 | NC_062327 |
| | | | | *Schizaphis graminum* (Rondani, 1852) | 15721 | NC_006158 |
| | | Macrosiphini | | *Acyrthosiphon pisum* (Harris, 1776) | 16971 | NC_011594 |
| | | | | *Acyrthosiphon caraganae* (Cholodkovsky, 1908) | 15933 | NC_064371 |
| | | | | *Aspidophorodon indicum* (David, Rajasingh & Narayanan, 1972) | 17161 | PQ468004 |
| | | | | *Brevicoryne brassicae* (Linnaeus, 1758) | 15749 | MW267824 |
| | | | | *Cavariella salicicola* (Matsumura, 1917) | 16317 | NC_022682 |
| | | | | *Diuraphis noxia* (Mordvilko, 1913) | 15784 | NC_022727 |
| | | | | *Indomegoura indica* (van der Goot, 1916) | 15220 | NC_045897 |
| | | | | *Lipaphis pseudobrassicae* (Davis, 1914) | 16743 | NC_072150 |
| | | | | *Macrosiphum rosae* (Linnaeus, 1758) | 15200 | NC_064372 |
| | | | | *Myzus persicae* (Sulzer, 1776) | 17382 | NC_029727 |
| | | | | *Neotoxoptera formosana* (Takahashi, 1921) | 15642 | NC_057970 |
| | | | | *Pterocomma pilosum* Buckton, 1879 | 12529 (partial) | KC840676 |

| | | | |
|---|---|---|---|
| | *Sitobion avenae* (Fabricius, 1775) | 15180 | NC_024683 |
| | *Uroleucon erigeronensis* (Thomas, 1878) | 15691 | MZ695840 |
| | *Uroleucon sonchi* (Linnaeus, 1767) | 17271 | MT533446 |
| Calaphidinae | *Appendiseta robiniae* (Gillette, 1907) | 15049 | MH643884 |
| | *Therioaphis trifolii* (Monell, 1882) | 16068 | MK766411 |
| Chaitophorinae | *Periphyllus diacerivorus* Zhang, 1982 | 16068 | MZ665537 |
| | *Periphyllus koelreuteriae* (Takahashi, 1919) | 16828 | PP856044 |
